# Global trends in research of endoscopic thyroidectomy from 2013 to 2022: a scientometric and visualization analysis

**DOI:** 10.3389/fendo.2023.1199563

**Published:** 2023-08-10

**Authors:** Daitian Zheng, Qiuping Yang, Jinyao Wu, Zhongming Zhou, Jiehui Cai, Lingzhi Chen, Zeqi Ji, Huiting Tian, Zhiyang Li, Yexi Chen

**Affiliations:** Department of Thyroid, Breast and Hernia Surgery, General Surgery, The Second Affiliated Hospital of Shantou University Medical College, Shantou, Guangdong, China

**Keywords:** endoscopic thyroidectomy, scientometric, Bibliometrix, CiteSpace, VOSviewer

## Abstract

**Introduction:**

Recently, endoscopic thyroidectomy has been developed and applied to thyroid surgery to achieve minimized neck scar formation and enhanced aesthetic outcomes. Our scientometric research in this paper offers a thorough overview of endoscopic thyroidectomy from 2013 to 2022.

**Methods:**

All pertinent articles on endoscopic thyroidectomy were obtained from the Web of Science Core Collection Database. The data on the number of citations and publications, most prolific countries and institutions, significant authors and journals, top themes, and keywords were analyzed by Biblioshiny, CiteSpace, and VOSviewer.

**Results:**

There were 758 publications, all of that were found from 2013 to 2022. The output of the annual publication showed an upward trend. A series of cases report by Anuwong et al. published in 2016 received the most citations. The country with the most articles published articles was South Korea, and the two countries with the most collaboration were South Korea and the United States. The most productive journal was *Surgical Endoscopy and Other Interventional Techniques*. Dionigi G, Kim HY, and Anuwong A were the writers with the most articles published, the highest h- and g-indices, and the strongest link strength, respectively. The keywords “endoscopic thyroidectomy”, “surgical”, “thyroidectomy”, “robotic thyroidectomy”, “experience”, and others were most used.

**Conclusion:**

The innovative surgical technique, transoral endoscopic thyroidectomy vestibular approach (TOETVA), leaves no scars and produces optimal cosmetic results. However, the long-term oncologic results for thyroid cancer performed with this approach are still missing. This scientometric analysis can offer valuable insights into the present research standing and key focal points in this domain, enabling researchers to gain a precise understanding of the state-of-the-art research in this area.

## 1 Introduction

With the growing utilization of neck ultrasonography and fine needle aspiration biopsy (FNAB), the prevalence of thyroid diseases has significantly increased globally. The results of the Global Cancer Statistics in 2020 showed that thyroid cancer ranks as the ninth most common malignancy worldwide, with a reported incidence of 586,202 new cases (1). Consequently, thyroid surgery has emerged as a prominent surgical intervention due to the growing incidence of thyroid disease. However, the conventional open thyroidectomy, which has been in use since the late 1800s, is associated with the occurrence of a prominent neck scar (2). This issue can potentially impinge upon the quality of life, especially for women who are highly sensitive to cosmesis (3).

In the pursuit of enhanced cosmetic outcomes and reduced neck scarring, the investigation of minimally invasive and remote-access approaches has gained considerable interest (4). The technique known as minimally invasive video-assisted thyroidectomy (MIVAT), which was initially proposed by Miccoli et al. in the late 1990s, has demonstrated favorable safety outcomes (5). MIVAT boasts several advantages over conventional open thyroidectomy, including reduced scar length and postoperative pain (6). However, it still involves a neck incision and leaves some scarring. With improvements in endoscopic technique, the proposition of performing endoscopic thyroidectomy to prevent anterior neck scarring became a reality. This resulted in the development of endoscopic thyroidectomy through different approaches, including the axilla (7), anterior chest (8), breast (9), retroauricular (10), and transoral approaches (11). These techniques provide patients with better results in terms of aesthetic outcomes and frequently express the various customs and expectations of patients from various regions (12). However, the adoption of these novel techniques was handled cautiously due to the technical challenges, doubts regarding oncological safety, and financial considerations (2). Additionally, there are no scientometric studies on endoscopic thyroidectomy.

To help researchers better comprehend the state of the research, we conducted a scientometric analysis of relevant papers in the field. We aim to identify the most prolific authors and countries, annual publication and citation information, prevalent topics, significant journals, and keyword analysis in the last 10 years to provide researchers with potential new directions of investigation.

## 2 Methods

### 2.1 Data collection

We conducted a comprehensive search of the science citation index expanded (SCI-E) (2003–present) database in the Web of Science Core Collection to obtain all relevant publications in the field of endoscopic thyroidectomy. The search strategies incorporated the Medical Subject Headings (MeSH) coupled with a diverse array of topic keywords to guarantee the accuracy and comprehensiveness of the retrieved data. A search query was conducted using the following formula: #1, TS = “thyroidectomy” OR TS = “thyroid surgery”; #2, TS = “endoscopic” OR TS = “endoscopy”; #3, “#1” and “#2”. Furthermore, only publications released from 2013 to 2022 were included in this filter of publications. Eight hundred fifty-four documents were found during the search, which was conducted on January 7th, 2023. Subsequently, we filtered the results by document type and language, considering only articles or reviews published in English, resulting in 774 publications comprising 660 articles and 114 reviews. A total of 80 documents, including 22 letters, 6 corrections, 11 meeting abstracts, 25 editorial materials, 12 non-English articles, and 4 non-English review papers, were excluded from the analysis. In addition, 16 early access publications were excluded from our analysis. Overall, 758 publications, including 111 review articles and 647 articles, were included in this scientometric study.

### 2.2 Data analysis

Using the “Analyze Results” feature of the WoS, we initially collected information on the publication years, authors, journals, document kinds, research areas, languages, countries, publishers, institutions, funding agencies, and so on. We also applied the “Citation Report” function of the WoS to obtain other data, such as the average citations per term, the number of citing articles (without self-citations and sum), the number of times cited (without self-citations and total sum), as well as the h-index.

The data, comprising document types, authors, institutions, languages, titles, keywords, abstracts, and cited references, underwent importation into CiteSpace, VOSviewer, and Biblioshiny, the last of which is a web-based interface for Bibliometrix.

The scientometric analysis of the collected data was carried out utilizing the R-tool Bibliometrix in R-studio (version 4.2.1). The generated results were visualized in a series of images (13). Through the importation of raw data files into the Biblioshiny website, essential details of the publications, including timespan, the number of documents, document types, authors, document contents, and author collaborations, were obtained. We used the data to make a preliminary assessment of whether the outcomes met the inclusive criterion. The amount of scholarly research produced annually, the most frequently cited articles and references, the production of countries and institutions, sources (the most relevant sources, sources impact, and source’ output over time), authors (the most contributing authors, author impact, and author’s output over time), and keywords were also included in the data set. The link between the most contributing authors, countries, and organizations was represented using a three-field plot. Hot studies were found using a network of keyword plus co-occurrences.

The identification and extraction of references and keywords with the strongest citation bursts were facilitated by CiteSpace (version 6.1.R6).

The analysis of most cited articles, co-occurrence analysis of keywords, and co-citation analysis of references were all performed by VOSviewer (version 1.6.18). In addition, the software was utilized to assess the degree of the connections between organizations, authors, and countries.

The statistical analyses were carried out using IBM SPSS statistics 26. For the representation of variables, both median with maximum and minimum values and mean with standard deviation were utilized for numerical variables. Meanwhile, categorical variables were expressed as frequencies and percentages. To verify the statistical significance and look for any correlations between the chosen variables, we employed Spearman’s correlation coefficient. When the *P* value of all tests is less than 0.05, it can be inferred that it is statistically significant.

**Figure 1:**
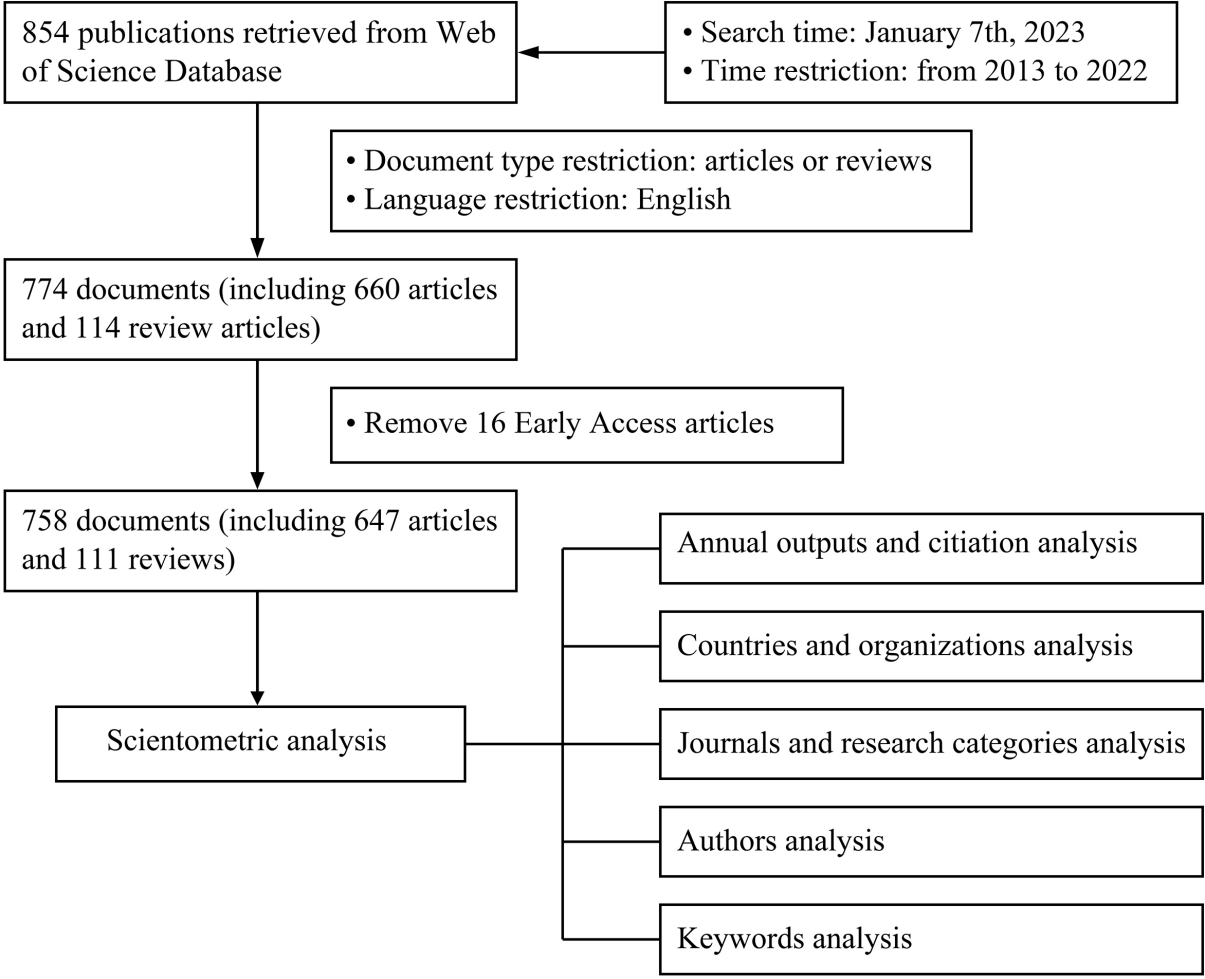
Literature collection and analysis flowchart.

A step-by-step process for gathering publications and assessing the findings is shown in **Figure 1**. Since this study made use of open-source data, the ethics committee’s permission was not required.

## 3 Results

### 3.1 Analysis of annual publications

**Figure 2:**
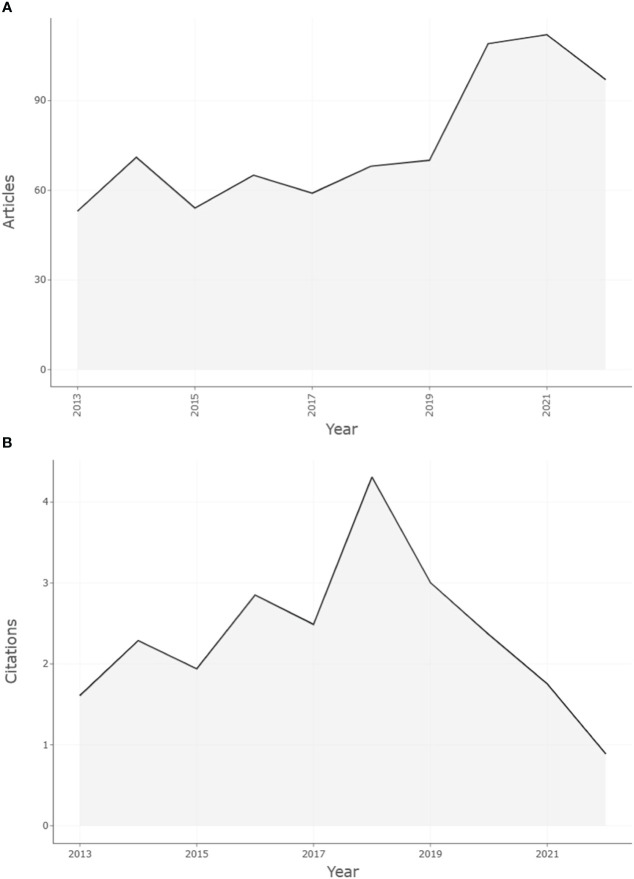
Number of articles published per year and average annual article citations from 2013 to 2022 in this research field. **(A)** The number of articles published per year. **(B)** Average annual article citations.

From the WoS, a total of 758 documents from 2013 to 2022 related to endoscopic thyroidectomy were retrieved. Overall, the annual publication volume showed an overall upward trend, increasing from 53 papers (7.00%) in 2013 to 97 papers (12.80%) in 2022, while the apex was in 2021 when 112 papers (14.78%) were published, as increasing in **Figure 2A** (*r* = 0.817; *p* < 0.005). From 2013 to 2019, the annual growth rate grew slowly and showed a fluctuating trend with a mean of 6.56%. It is worth noting that there was a sudden increase in annual publication output in 2020 (growth rate of 55.71%) which exceeded 100. However, the annual publication growth rate decreased in 2021 (2.75%) and even became negative in 2022 (-13.39%). The median annual production growth rate was 2.94%, with a maximum in 2020 (55.71%) and a minimum in 2015 (-23.94%).

### 3.2 Citation analysis

Of the total relevant publications, there were 2,546 citing articles, of which 1,986 (78.00%) were not self-citations, and 560 were self-citations. The articles were cited 8,887 times, including 4,295 without self-citation, accounting for 48.33%. In addition, the average citation count per article was 11.72. In **Figure 2B**, the average number of citations showed a fluctuating pattern between 2013 and 2017. Notably, the average citation count increased significantly in 2018, and it was the highest number of years (4.31). However, 2018 to 2022 shows a decreasing trend.

**Supplementary Table S1**, which was visualized using VOSviewer software, lists the foremost ten most cited publications within the scope of endoscopic thyroidectomy research. Highly cited publications can point us to the frontiers of research. All these studies were released between 2013 and 2018. The largest percentages were published in 2014 and 2018 (both were 30%). The most cited articles were written by Anuwong A et al. (14), with the paper entitled “Transoral Endoscopic Thyroidectomy Vestibular Approach: A Series of the First 60 Human Cases”, which received 272 immediate citations (14). The work “Safety and Outcomes of the Transoral Endoscopic Thyroidectomy Vestibular Approach” by Anuwong A et al. (15) was cited 214 times (15). The third article was “Transoral endoscopic thyroidectomy vestibular approach (TOETVA): indications, techniques and results” by Anuwong A et al. (16), which was cited 134 times (16). Two publications received more than 200 citations, representing 0.26% of all citations, while six (0.79%) papers received more than 100 citations and 27 (3.56%) more than 50 times. Notably, over the previous three years, the article with the highest citation count (Anuwong A et al., (14) received 167 citations, representing 61.40% of all citations attributed to it, while the second most frequently cited article (Anuwong A et al., (15) was cited 165 times, accounting for 77.10% of all its citations.

**Figure 3:**
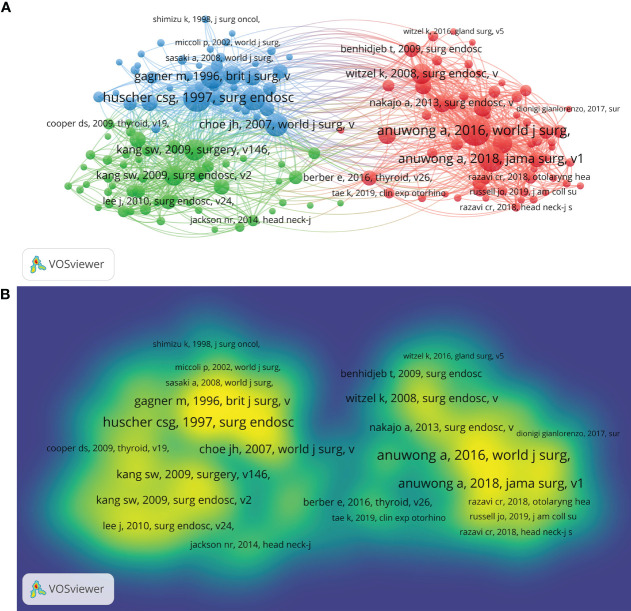
Co-citation analysis of references in documents cited over 20 times. **(A)** Co-citation network map between cited references. **(B)** Mapping of the frequency and intensity of co-citation of references. Yellow indicates the highest frequency of citations.

To analyze the co-citation network of the references cited in these publications, a citation threshold of at least 20 citations was used, resulting in 154 references being considered in the analysis (**Figure 3**). Larger nodes in **Figure 3A** indicate a larger number of references. The more co-references, the more yellow the color (**Figure 3B**). The references cited in **Figure 3A** have three clusters, with the most prominent research field indicated in red in the top cluster comprising 61 items. In **Figure 3**, Anuwong A’s article (14) was cited the most, reaching 184 times, which indicates the great value of this reference (14). The top five most frequently cited references were Anuwong A (2016) (184 citations) (14), Hüscher CS (17) (160 citations) (17), Anuwong A (2018) (145 citations) (15), Gagner M (1996) (125 citations) (18), and Choe JH (19) (121 citations) (19). **Supplementary Figure S1** depicts the top 25 references which exhibit the most powerful citation bursts identified by CiteSpace. Notably, the highest number of them developed citation bursts was in 2013 (17/25, 68%), followed by 2020 (3/25, 12%), 2016 (2/25, 8%), and 2018 (2/25, 8%). Moreover, three references (12%) had citation bursts lasting through 2022. The article by Kang SW, which had citation bursts from 2013 to 2017, displayed the strongest citation burstness (strength = 20.62) (20).

### 3.3 Analysis of countries

**Figure 4:**
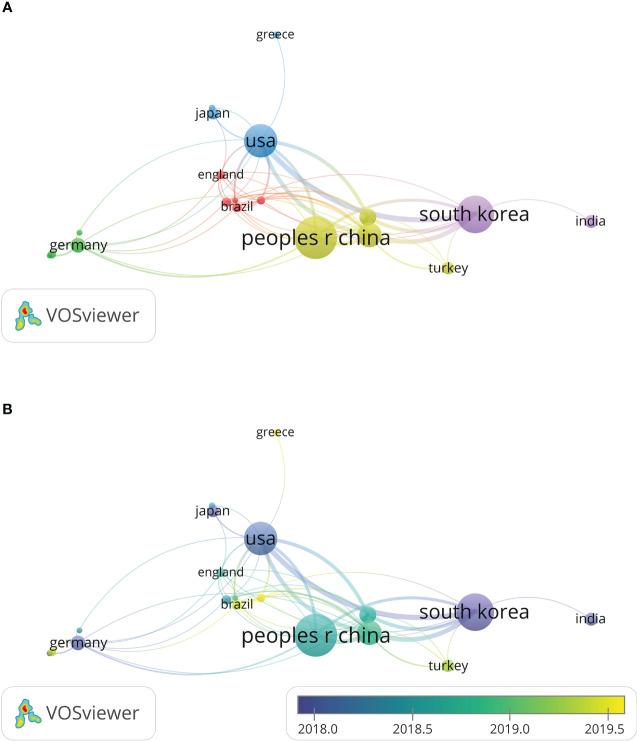
Co-authorship analysis of countries that have published over five articles. **(A)** Co-authorship network map of countries. **(B)** Co-authorship overlay map of countries (blue: earlier, yellow: later).

A total of 42 countries and regions, both developed and developing, were represented in this research area. China demonstrated the highest level of productivity with the greatest number of publications in this field (n = 260, 34.30%). It was followed by South Korea (n = 167, 22.03%), the United States (n = 96, 12.66%), Italy (n = 39, 5.15%), and India (n = 23, 3.03%). **Table 1** shows the top ten countries received the most citations, with South Korea being the most cited country (n = 2,881), followed by China (n = 1,858), the United States (n =1,522), Thailand (n=834), Italy (n = 386). The co-authorship analysis included 23 countries with over five publications in this field. The United States exhibited the highest total link strength (131), Italy (128), South Korea (124), and Thailand (79), China (76) (**Figure 4**). A smaller distance between the circles indicates a stronger relationship between countries based on the frequency of co-occurrence, and a larger circle indicates a reflection of the strength of the total linkage of countries. A few developing nations started to stand out in this regard, such as Thailand (total link strength = 79), China (76), and Brazil (15). The Bibliometrix package was used to determine the level of cooperation between the nations/regions in **Table 2**. The two countries that collaborated most frequently were South Korea and the United States.

**Table 1:** The top 10 countries received the highest number of citations.

Country	Total production	Total citations	Average Article Citations
South Korea	167	2,881	17.25
China	260	1,858	7.15
the United States	96	1,522	15.85
Thailand	15	834	55.60
Italy	39	386	9.90
Germany	20	318	15.90
Japan	17	210	12.35
United Kingdom	6	135	22.50
India	23	110	4.78
Turkey	20	77	3.85

**Table 2:** The collaboration between countries or regions.

From	To	Frequency
Korea	the United States	41
China	Italy	32
South Korea	Italy	32
the United States	Italy	26
China	South Korea	21
China	the United States	21
South Korea	Thailand	21
Italy	Thailand	20
the United States	Thailand	20
China	Thailand	8

### 3.4 Institutions analysis

**Figure 5:**
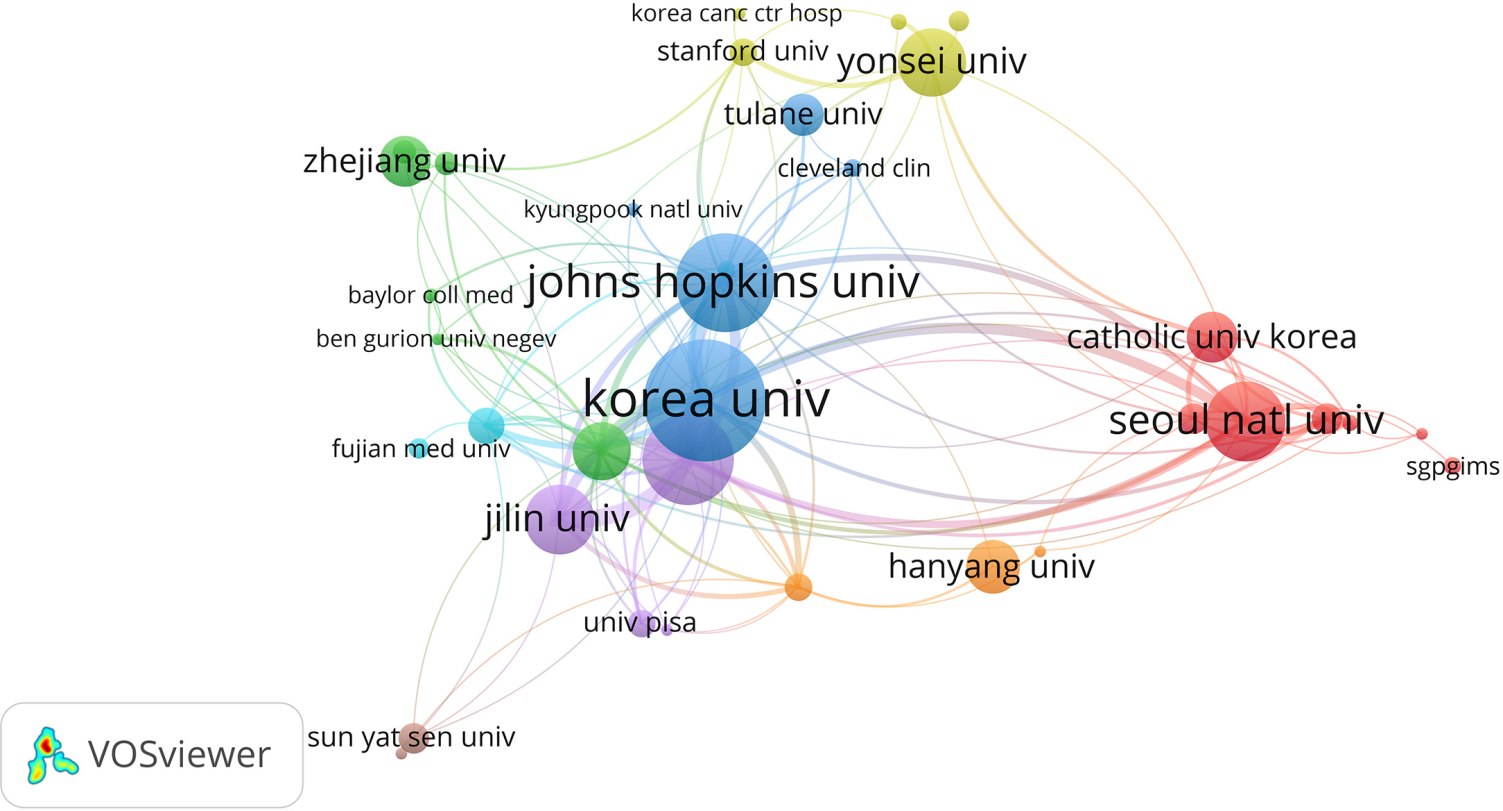
Co-authorship network map of organizations.

Of these 758 publications, 724 institutions contributed to this area of work. The institution that contributed the most to the number of publications was Korea University (n = 52, 6.86%), followed by Johns Hopkins University (n = 42, 5.54%), the University of Messina (n = 39, 5.15%), the Seoul National University (n = 34, 4.49%), and the Jilin University (n = 30, 3.96) (**Table 3**, **Figure 5**). After that, the co-authorship of organizations with more than five papers was analyzed, obtaining 55 institutions. Analysis of the total link strength for such institutions revealed that the Korea University (118), the University of Messina (95), Johns Hopkins University (82), the Seoul National University (64), and Jilin University (58) constituted the top five institutions.

**Table 3:** The top 10 productive institutions in this research field.

Organization	Total production	Total citations	Total link strength
Korea University	52	1,275	118
Johns Hopkins University	42	932	82
University of Messina	39	601	95
Seoul National University	34	580	64
Jilin University	30	270	58
Yonsei University	29	621	19
Police General Hospital	25	967	60
Hanyang University	23	491	4
Catholic University Korea	22	311	18
Zhejiang University	22	229	5

### 3.5 Journals analysis

**Figure 6:**
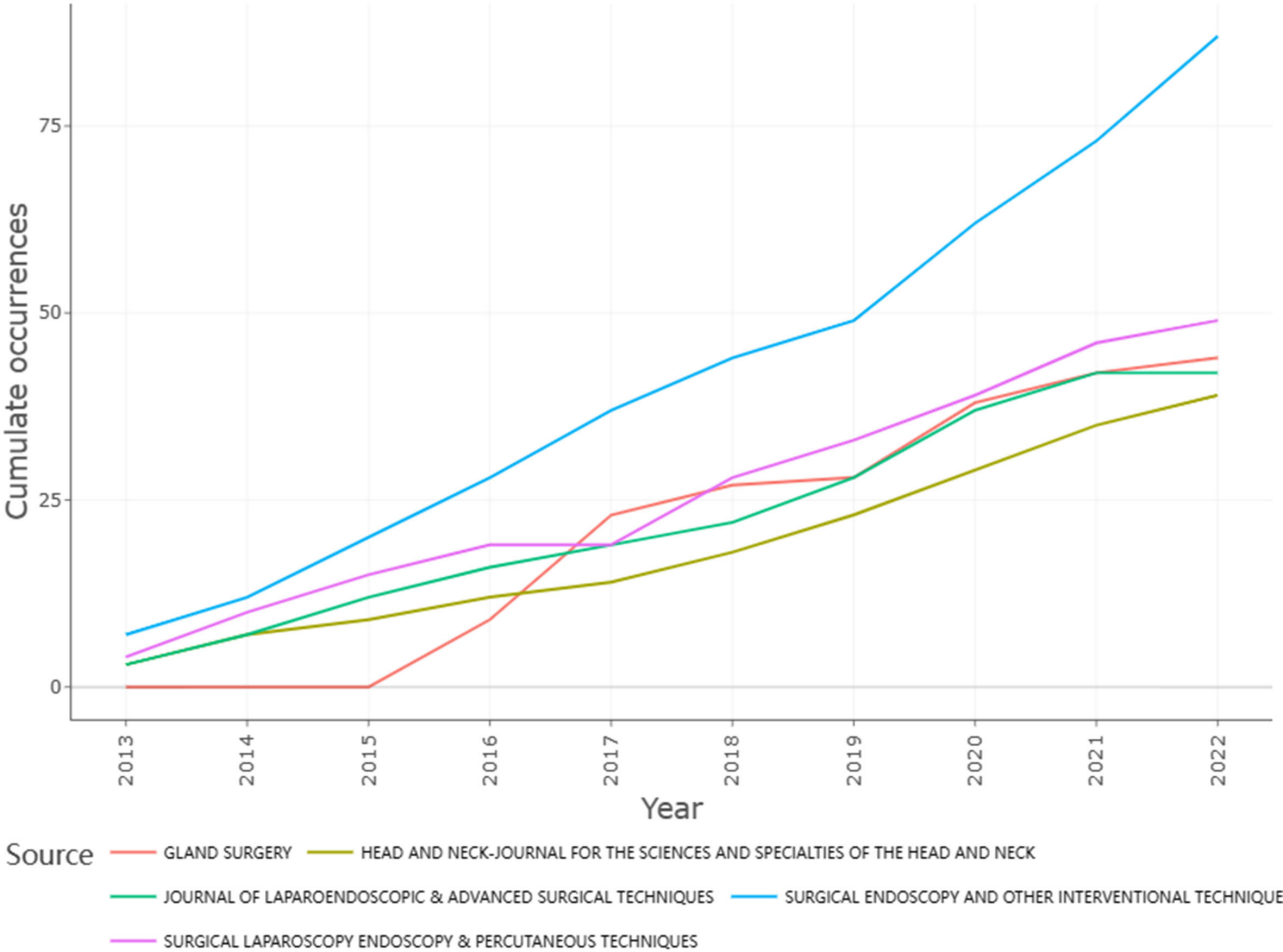
Source growth over the years in this field.

In 169 journals, these 758 papers were released. The top 10 journals with the highest number of published articles are presented in **Supplementary Table S2**. There were 339 papers published in these top 10 journals, accounting for 44.72% of all papers published. Most documents were published in *Surgical Endoscopy and Other Interventional Techniques* (n = 87). With 49 publications, *Surgical Laparoscopy Endoscopy & Percutaneous Techniques* was the second most popular journal. The third was *Gland Surgery* (n = 44), followed by the *Journal of Laparoendoscopic & Advanced Surgical Techniques* (n = 42) and *Head and Neck-Journal for the Sciences and Specialties of the Head and Neck* (n = 39). These journals mainly focus on surgery. With 2,384 citations, *Surgical Endoscopy and Other Interventional Techniques* was the most cited journal, followed by *World Journal of Surgery* (1,488 citations), *Surgery* (995 citations), *Surgical Laparoscopy Endoscopy & Percutaneous Techniques* (803 citations), and *Head and Neck-Journal for the Sciences and Specialties of the Head and Neck* (773 citations). The assessment of the impact of these journals was based on the application of the h-index, which is a metric to quantify their significance (21). The journal *Surgical Endoscopy and Other Interventional Techniques* had the largest h-index of 20, followed by *Head and Neck-Journal for the Sciences and Specialties of the Head and Neck* (h-index = 14), *Journal of Laparoendoscopic & Advanced Surgical Techniques* (h-index = 14), *World Journal of Surgery* (h-index = 13), *Gland Surgery* (h-index = 11), and *Surgical Laparoscopy Endoscopy & Percutaneous Techniques* (h-index = 11) (**Table 4**). As shown in **Figure 6**, the *Journal of Laparoendoscopic & Advanced Surgical Techniques*, *Gland Surgery*, *Surgical Endoscopy and Other Interventional Techniques*, and *Head and Neck-Journal for the Sciences and Specialties of the Head and Neck* have all been active in this area over time.

**Table 4:** The influence of the top 10 journals published in this field.

Element	h-index	g-index	m-index	TC	NP	PY-start
Surgical Endoscopy and Other Interventional Techniques	20	34	1.818	1471	87	2013
Head and Neck-Journal for the Sciences and Specialties of the Head and Neck	14	24	1.273	638	39	2013
Journal of Laparoendoscopic & Advanced Surgical Techniques	14	21	1.273	532	42	2013
World Journal of Surgery	13	24	1.182	594	26	2013
Gland Surgery	11	21	1.375	517	44	2016
Surgical Laparoscopy Endoscopy & Percutaneous Techniques	11	16	1.000	359	49	2013
Annals of Surgical Oncology	10	11	0.909	320	11	2013
Otolaryngology-Head and Neck Surgery	8	11	0.800	303	11	2014
Thyroid	8	12	0.727	301	12	2013
Annals of Surgical Treatment and Research	7	10	0.700	157	10	2014

TC, total citations; NP, number of publications; PY-start, publication year start.

### 3.6 Research category analysis

There were 42 research categories involved in the study. The top five subject categories are presented in **Table 5**, ascertained on the basis of the number of publications: The main subject category was “Surgery” with a publication count of 498, followed by “Otorhinolaryngology” (133 publications), “Oncology” (74 publications), “Endocrinology Metabolism” (51 publications), and “Medicine General Internal” (35 publications). Regarding the publishers, “Springer Nature” (214 publications), “Wiley” (81 publications), “Elsevier” (72 publications), “Lippincott Williams & Wilkins” (67 publications), and “MDPI” (54 publications) had the highest number of publications.

**Table 5:** The five most prominent categories in this field.

Categories	Document	Count
Surgery	498	65.699%
Otorhinolaryngology	133	17.546%
Oncology	74	9.763%
Endocrinology Metabolism	51	6.728%
Medicine General Internal	35	4.617%

### 3.7 Authors analysis

**Figure 7:**
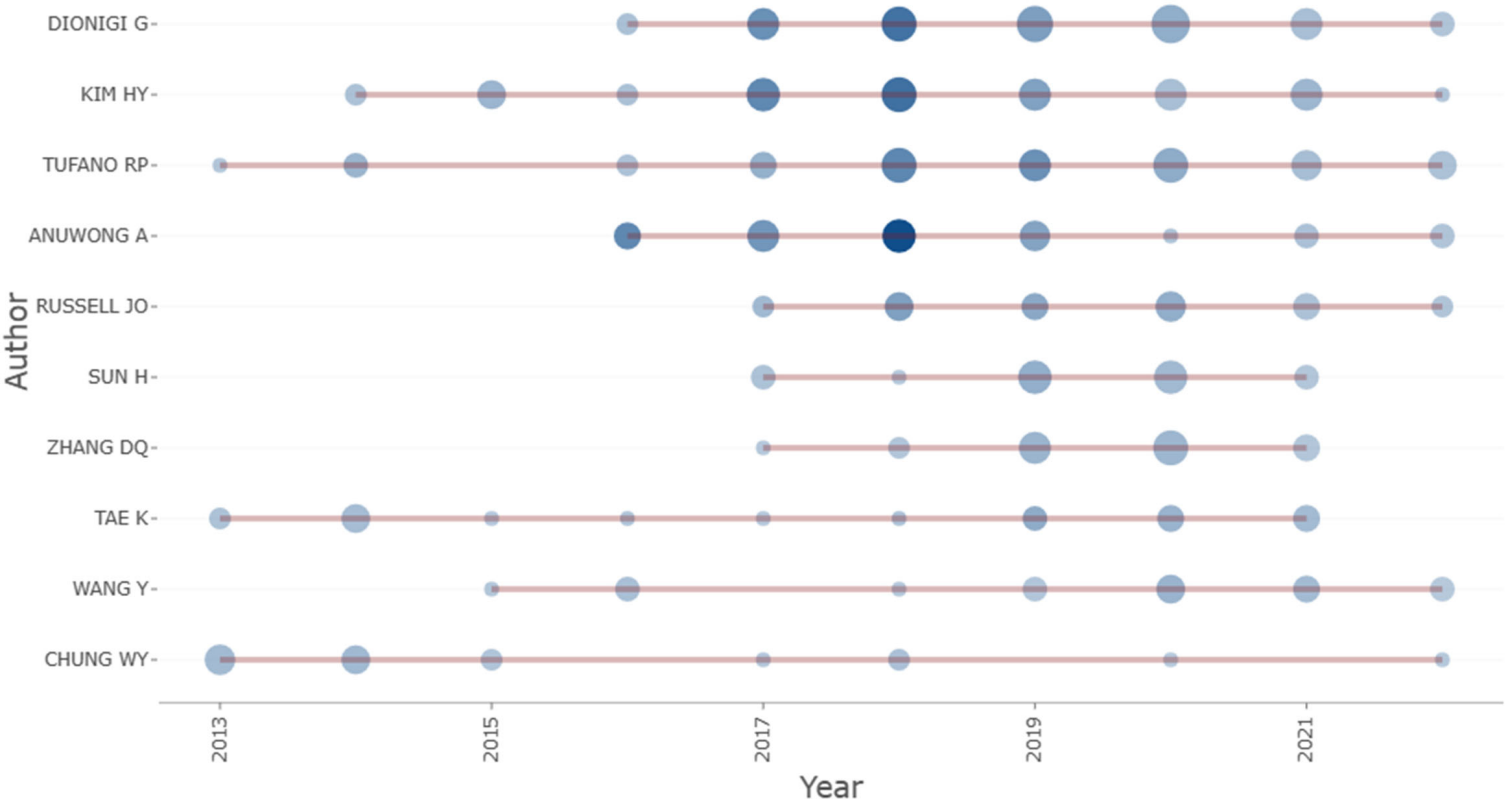
Authors’ output of this field over time.

There were 2,595 authors in our dataset, including ten authors of single-authored papers and 2,585 authors were associated with multiauthored papers. While there were 2,595 authors overall, there were 4,556 total author appearances. The number of single-authored documents was 13. There were 3.42 authors per document on average and 6.01 co-authors per document on average. The most productive author, Dionigi G, contributed to 50 publications, which was 6.60% of all publications. Kim HY ranked second with 48 publications (6.32%), followed by Tufano RP (n = 46, 6.07%) and Anuwong A (n = 32, 4.22%) (**Table 6**). There was greater importance and centrality to the more productive authors in this field. A total of eight authors published over 20 articles, while 31 authors authored more than ten articles. Notably, there are 1,518 authors (58.50%) who have recently begun publishing relevant articles in the last five years. **Figure 7** displays the annual citation count, with darker coloration indicative of a higher total citation count. Meanwhile, the size of the dots is proportional to the number of publications. Most of Chung WY’s articles were released in the first five years, whereas the majority of Dionigi G and Tufano RP’s articles were released in the last five years. Tae K and Wang Y generally kept up a steady trend in publishing articles.

**Table 6:** Top 10 authors in terms of the number of published articles.

Authors	Articles	Articles Fractionalized	h-index	g-index	m-index	TC	NP	PY-start
Dionigi G	50	6.85	19	31	2.375	1,057	50	2016
Kim HY	48	7.52	22	34	2.200	1,270	48	2014
Tufano RP	46	7.38	21	31	1.909	1,042	46	2013
Anuwong A	32	6.37	21	32	2.625	1,452	32	2016
Russell JO	23	3.45	13	22	1.857	525	23	2017
Sun H	23	3.07	9	13	1.286	215	23	2017
Zhang DQ	23	3.14	8	12	1.143	176	23	2017
Tae K	22	5.68	14	22	1.273	490	22	2013
Wang Y	20	3.30	14	22	1.273	490	22	2013

**Supplementary Table S3** shows the top 5 most cited authors. The top cited author was Anuwong A, with 1,425 citations, followed by Kim HY (1,149 citations), Tufano RP (1,041 citations), Dionigi G (1,036 citations), and Richmon JD (566 citations). In terms of the h-index, Kim HY led the pack with an h-index of 22. Anuwong A and Tufano RP, each with an h-index of 21, secured the second position, while Dionigi G’s h-index was measured at 19. Tae K and Wang Y followed with an h-index of 14. In total, eight authors who published the highest number of articles had an h-index greater than 10. By correcting the h-index for time, the m-index helps identify scientists who are truly successful based on their seniority (21). The highest m-index belongs to Anuwong A with 2.625, followed by Dionigi G (m-index = 2.375), Kim HY (m-index = 2.200), Tufano RP (m-index = 1.909), and Russell JO (m-index = 1.857). In total, 112 authors who published more than five articles were analyzed.

**Figure 8:**
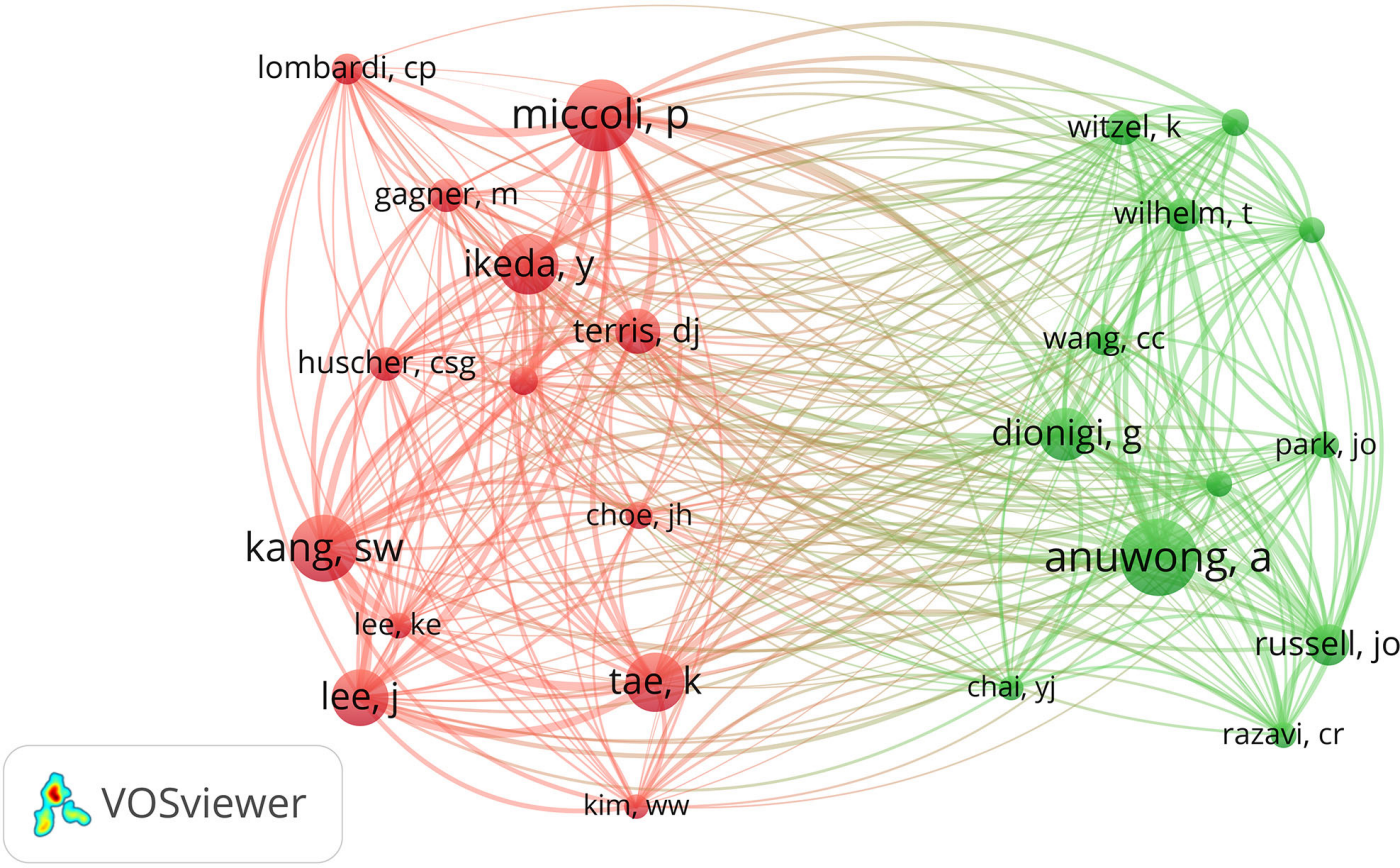
Co-citations network map of authors received over 100 citations.

Using VOSviewer software, we analyzed co-citations between authors (**Figure 8**). There were 25 authors analyzed with over 100 citations. In the co-citation analysis, the results indicated that Anuwong A exhibited the highest link strength (total link strength = 3,889). Miccoli ranked second in total link strength with 3,726. In the subsequent ranks, Kang, Tae, and Ikeda respectively demonstrated link strengths of 3,265, 2,770, and 2,708.

In **Supplementary Figure S2**, the three fields plot showing the relationships between top authors (left), the most productive countries (center), and institutions (right). The connections and contributions of a country, author, and institution are indicated by the thickness of lines and the height of nodes, respectively. The three countries with the most connections were South Korea, China, and the United States. Seoul National University and Korea University made the largest contributions among South Korean organizations. Dionigi G, Kim HY, and Tufano RP exhibited the highest degree of international collaboration.

### 3.8 Analysis of keywords

**Figure 9:**
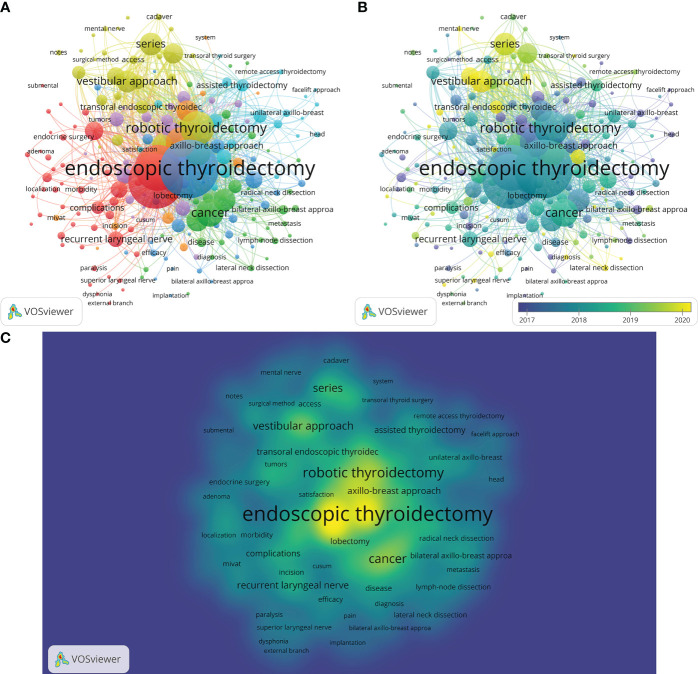
Co-occurrence analysis of keywords. **(A)** Network mapping of keywords (10 clusters). **(B)** Overlay mapping of keywords. **(C)** Density mapping of keywords.

With the help of VOSviewer, we analyzed 210 keywords that occurred more than five times. For those keywords with high relevance, the possibility of grouping them into the same cluster with the same color is higher. These chosen keywords were loosely grouped into seven clusters, as seen in **Figure 9A**. The keywords with the same frequency were performed by the density visualization map (**Figure 9C**). The most frequent appearing keywords were “endoscopic thyroidectomy” (n = 322), “surgery” (n = 289), “thyroidectomy” (n = 223), “robotic thyroidectomy” (n = 142), “experience” (n = 117), and so on. Moreover, as illustrated in the overlay visualization map (**Figure 9B**), the temporal distribution of these keywords is represented by color. The later the keyword appears, the more yellow the color is. Over time, the research emphasis on surgical techniques has evolved from transaxillary endoscopic thyroidectomy and video-assisted thyroidectomy to robotic surgery and then come the transoral approach.

The term “keywords plus” refers to words and phrases which are absent from the title of the article but frequently present in the titles of cited references (22). **Supplementary Figure S3** consists of four quadrants, each containing words or phrases with various characteristics. The first quadrant (motor themes) consists of terms that are highly centralized and dense, indicating their significance and well-development in this research area. The second quadrant (niche themes) comprises keywords with high density and low centrality, making them well-developed but less important. The third quadrant (emerging or declining themes) consists of poorly centralized and dense keywords with weak links between the themes in this research field. They might be emerging right now, or they might be going away. The fourth quadrant (basic themes) contains basic concepts and knowledge with high centrality but low density in this area of study. The fourth quadrant (Basic themes) contains basic knowledge and concepts with high centrality but low density in this area of study (22). As shown in **Supplementary Figure S3**, the keywords plus were categorized into three clusters in different colors. Cluster 1 (red) was the keywords (“surgery”, “series”, and “vestibular approach”) in the motor theme of this study area, indicating that these keywords were well-developed and significant. The keywords “endoscopic thyroidectomy”, “surgery”, and “experience” in cluster 2 (blue) were located between the Niche themes and the declining theme Emerging or declining themes, and cluster 3’s (green color) keywords (“surgery”, “endoscopic thyroidectomy”, “experience”) were located between declining theme Emerging or declining themes and the merging or lining theme quadrant in this research area.

In the references, some of these keywords were displayed as citation bursts. In general, burst words are those that are cited frequently over time. Burst keywords were identified to indicate frontier topics in the field by CiteSpace. **Supplementary Figure S4** presents a list of the top 25 keywords with the highest citation bursts. The leading eleven keywords indicated emerging trends in endoscopic thyroidectomy research from 2013-2017. Between 2015 and 2018, the keywords “complications”, “retroauricular approach”, and “natural orifice surgery” became active. The latter eleven keywords have garnered significant attention recently, indicating that they are the hotspots of current research. In addition, the keywords whose citation burst remains until 2022 (“series”, “vestibular approach”, “quality of life”, “approach TOETVA”, “transoral endoscopic thyroidectomy”, and “impact”) are probably the most popular topics. From 2018-2022, the keyword “series” had the strongest citation bursts (strength = 10.84).

## 4 Discussion

This study conducted a scientometric analysis to investigate the scientific literature on endoscopic thyroidectomy during the period of 2013 to 2022, utilizing Bibliometrix, VOSviewer, and CiteSpace. A total of 758 documents were identified as of 2022. The research output was noted to be prolific between 2013 and 2022, with the greatest number of publications occurring in 2021. The annual growth rate of publication production reached its peak in 2020, denoting a sudden surge of interest in this area, followed by a recent downturn. The identified articles garnered 2,546 citations overall, averaging 11.72 citations per document. The most published papers in the top 10 citations were in 2014 and 2018, both with 30%. They described the available surgical approaches to endoscopic thyroidectomy and their outcome. It is worth noting that seven of these papers focus on TOETVA (14–16, 23–26), which indicated that TOETVA was a hot topic of research in this area of thyroidectomy. It is probably due to the scarless and cosmetic advantages of TOETVA, although its safety and efficacy are still being discussed. There is no doubt that Anuwong A played an important role in the study of TOETVA, as the top three cited articles were authored by him and pointed out the safety, indications, techniques, and results of TOETVA (14–16). In addition, one article published in “surgery” compared the TOETVA with endoscopic thyroidectomy by the areola approach and found that TOETVA has the advantages of a short surgical route, minimal trauma, rapid postoperative wound healing, and no visible residual surgical scarring (24). Although TOETVA is an ideal minimally invasive thyroidectomy, technical barriers have prevented the widespread dissemination of this procedure (26). New surgical techniques are constantly being described. Notably, a meta-analysis has demonstrated that robotic thyroidectomy is comparable to conventional open and endoscopic thyroidectomy in terms of safety, feasibility, and effectiveness while offering enhanced cosmetic satisfaction compared to traditional thyroidectomy. Besides, robotic thyroidectomy has many advantages, such as enhanced access and visualization and reduced transfer to instrumentation tremors (27).

This research field has received contributions from 42 countries. China had the maximum quantity of publications; South Korea had the maximum quantity of citations, and the United States exhibited the greatest link strength with other nations. Of the five most productive institutions, two were located in South Korea. In addition, South Korea also boasts the largest number of organizations within the top five regarding total link strength, with two. There is no doubt that the contributions of China, South Korea, and the United States to this area of study are indisputable. Developed countries have contributed more to this area, but over time, some developing countries have begun to emerge in this area. For instance, two of the top five and four of the top ten most productive nations are both in the developing world, which could be highly encouraging for researchers in those countries.

Out of the 614 types of journals considered, six of the top 10 most productive ones were classified in the field of “surgery”, while the other four were categorized as “otorhinolaryngology” (n = 3) and “endocrinology and metabolism” (n = 1). *Surgical Endoscopy and Other Interventional Techniques*, *Surgical Laparoscopy Endoscopy & Percutaneous Techniques*, *Gland Surgery*, and *Journal of Laparoendoscopic & Advanced Surgical Techniques* consistently exhibited exceptional publication output in this field, signifying the importance of the surgery and medicine sciences.

The retrieved papers were published with contributions from 10,339 authors. Among these authors, Dionigi G (n = 50, 6.60%), Kim HY (n = 48, 6.32%), Tufano RP (n = 46, 6.07%), Anuwong A (n = 32, 4.22%), and Russell JO (n = 23, 3.03%), emerged as the top 5 most prolific contributors. Notably, Kim HY demonstrated outstanding research acumen by garnering the highest h-index and g-index. Anuwong A, the strongest link strength author, published a report on their initial TOETVA experience in 2016, which involved a series of 60 patients with minimal complications (14). Furthermore, two articles published by Anuwong A in 2018 reported larger numbers of TOETVA case series with encouragingly excellent results, confirming that TOETVA is the preferred transoral neck access technique (15, 16). The most cited paper was a case series report he published in 2016, which was cited 2,766 times. He established a cornerstone in this field of research, contributing significantly to the refinement of the surgical technique of transoral endoscopic thyroidectomy and validating its safety.

From the keyword analysis, the exploration of the approach to endoscopic thyroidectomy has been the focus of this research area. Among the approaches, the axillary approach was the first to become a research hotspot in a decade, followed by the retroauricular approach. Recently, the research hotspot was shifted to transoral endoscopic thyroidectomy. The top 25 most frequently cited keywords reveal a trend toward exploring new surgical techniques with smaller incisions and less trauma. In 2000, Ikeda Y et al. successfully performed the first transaxillary endoscopic thyroidectomy, placing the surgical incision more discreetly in the axilla and avoiding scarring of the neck and chest skin (28). The gasless axillary approach is usually used, effectively avoiding characteristic postoperative complications such as hypercapnia and providing excellent exposure and a good surgical view (29). However, clearance of the contralateral lesions and lymph nodes is more difficult and therefore requires further study. The first retroauricular thyroidectomy was performed by Singer MC et al. utilizing a surgical robot and was mainly promoted by South Korean teams, such as Tae, Koh, and Jung (30–33). The retroauricular approach uses an occipital hairline and retroauricular incision, which has an incision closer to the thyroid gland and a smaller anatomical area than the axillary approach (34, 35). It offers cosmetic advantages with scars hidden under the hair and auricle. However, the restricted working space and the difficulty of accessing the contralateral thyroid lobe through a unilateral incision limit its popularity (33). The focus of the current study, transoral endoscopic thyroidectomy, concerns sublingual and oral vestibular approaches (24, 36). Witzel K et al. (37) first attempted the sublingual approach, but it was not popular due to the high complication rate. TOETVA was first described by Richmon JD et al. (38) and was refined by Anuwong A and colleagues (14). Compared to other techniques, TOETV offers superior field views, making level VII central neck dissection simple to complete (39). Moreover, the transoral approach is less intrusive in the working space than other approaches and avoids skin scars (29). Although its application in both benign and malignant thyroid lesions is considered feasible, long-term oncological results regarding its use in thyroid cancer are still lacking (12). Therefore, further studies with sufficient follow-up are required to assess its safety, particularly with regard to technical viability and oncological integrity.

Our study does have certain restrictions. First, because it is challenging to combine the results of different databases directly owing to the restrictions of scientometric software, we just searched the WoS instead of merging it with the results from other search databases like Scopus, PubMed, or Embase. Not all journals in any field are included on the WoS. In addition, it lacks the representation of non-English journals because of the focus on English journals. Despite this, the most popular literature database for scientometric investigations is still the WoS. It is the most user-friendly and straightforward tool with features for primary analysis like “create citation report” and “analyze results”. The WoS format was generally recognizable by bibliometric tools. It is impossible to obtain all of an article’s details, such as the nationality of the author. However, this problem can be solved by manual inquiry into the author (40). Screening articles requires relevant criteria or the combined opinion of numerous experts, as the subjective nature of the screening criteria. Furthermore, the journals that garner a significant number of citations may possess a multidisciplinary focus and encompass a broad spectrum of research areas, which results in inadequate specificity to our field. Additionally, such articles may have been published in the distant past and may not reflect the most recent advances in the field, thus diminishing their citation impact. Nevertheless, we anticipate that our study will inspire more work in similar disciplines.

## 5 Conclusion

In this study, we presented a scientometric evaluation of the retrieved publications on endoscopic thyroidectomy published between 2013 and 2022. Our analysis revealed that the production of documents related to this topic has increased rapidly since 2013. Among the countries that contributed to the literature, South Korea was the most published country. Moreover, the two countries with the most collaboration were South Korea and the United States. Notably, *Surgical Endoscopy and Other Interventional Techniques*, *Surgical Laparoscopy Endoscopy & Percutaneous Techniques*, and *Gland Surgery* were three of the most fruitful journals. As for the authors, Dionigi G, the most prolific author, and Anuwong A exhibited the highest link strength. At the same time, Kim HY had achieved the highest h-index and g-index. Additionally, the keyword analysis revealed that certain areas require further attention, such as “transoral endoscopic thyroidectomy”, “vestibular technique”, “quality of life”, and “complications”. It is worth mentioning that TOETVA is the only technique that is entirely scarless. Nevertheless, there are still a few technical and oncologic issues regarding this approach. Thus, more research and proper follow-up are required to determine its safety, particularly in cases of malignancy. Given the heavy burden of thyroid cancer today, interest in endoscopic thyroidectomy will continue to grow, thereby contributing to further advances in this field.

## Data availability statement

The original contributions presented in the study are included in the article/**Supplementary Material**. Further inquiries can be directed to the corresponding authors.

## Author contributions

DZ conceived and designed the study, conducted the visual analysis of the data, and wrote the initial draft. QY and JW retrieved the dataset from the Web of Science and provided software guidance, while ZZ, JC, and LC revised the second draft. ZJ, HT, ZL, and YC were responsible for interpreting the findings of the study. All authors contributed to the article and approved the submitted version.

## References

[1] Sung H Ferlay J Siegel RL Laversanne M Soerjomataram I Jemal A. Global cancer statistics 2020: GLOBOCAN estimates of incidence and mortality worldwide for 36 cancers in 185 countries. CA Cancer J Clin (2021) 71(3):209–49. doi: 10.3322/caac.21660 33538338

[2] Kudpaje A Subash A Subramaniam N Palme CE Us VR Arakeri G. Remote access thyroid surgery: A review of literature. Indian J Surg Oncol (2022) 13(1):191–8. doi: 10.1007/s13193-021-01364-y PMC8986942 35462662

[3] Liu YH Xue LB Zhang S Yang YF Li J. Appearance characteristics of incision, satisfaction with the aesthetic effect, and quality of life in of thyroid cancer patients after thyroidectomy. Int J Health Plann Manage (2021) 36(3):784–92. doi: 10.1002/hpm.3111 33502801

[4] de Vries LH Aykan D Lodewijk L Damen JAA Borel Rinkes IHM Vriens MR. Outcomes of minimally invasive thyroid surgery - A systematic review and meta-analysis. Front Endocrinol (Lausanne) (2021) 12:719397. doi: 10.3389/fendo.2021.719397 34456874 PMC8387875

[5] Miccoli P Fregoli L Rossi L Papini P Ambrosini CE Bakkar S. Minimally invasive video-assisted thyroidectomy (MIVAT). Gland Surg (2020) 9(Suppl 1):S1–5. doi: 10.21037/gs.2019.12.05 PMC6995905 32055492

[6] Kim K Kang SW Kim JK Lee CR Lee J Jeong JJ. Surgical outcomes of minimally invasive thyroidectomy in thyroid cancer: comparison with conventional open thyroidectomy. Gland Surg (2020) 9(5):1172–81. doi: 10.21037/gs-20-512 PMC7667120 33224792

[7] Xu S Yang Z Guo Q Zou W Liu S Gao Q. Surgical steps of gasless transaxillary endoscopic thyroidectomy: From A to Z. J Oncol (2022) 2022:2037400. doi: 10.1155/2022/2037400 36536786 PMC9759389

[8] Yang Y Sun D Yang J Chen J Duan Y. Endoscopic thyroidectomy in anterior chest approach versus open thyroidectomy for patients with papillary thyroid carcinomas, a retrospective study. J Laparoendosc Adv Surg Tech A (2020) 30(5):488–94. doi: 10.1089/lap.2019.0694 32182158

[9] Yan HC Xiang C Wang Y Wang P. Scarless endoscopic thyroidectomy (SET) lateral neck dissection for papillary thyroid carcinoma through breast approach: 10 years of experience. Surg Endosc (2021) 35(7):3540–6. doi: 10.1007/s00464-020-07814-y 32691204

[10] Dong F Yang A Ouyang D. Retroauricular single-site endoscopic thyroidectomy-A balanced endoscopic approach for thyroid excision. JAMA Surg (2023) 158(5):548–9. doi: 10.1001/jamasurg.2022.7723 36753130

[11] Jongekkasit I Jitpratoom P Sasanakietkul T Anuwong A. Transoral endoscopic thyroidectomy for thyroid cancer. Endocrinol Metab Clin North Am (2019) 48(1):165–80. doi: 10.1016/j.ecl.2018.11.009 30717900

[12] Rossi L Materazzi G Bakkar S Miccoli P. Recent trends in surgical approach to thyroid cancer. Front Endocrinol (Lausanne) (2021) 12:699805. doi: 10.3389/fendo.2021.699805 34149628 PMC8206549

[13] Aria M Cuccurullo C. bibliometrix: An R-tool for comprehensive science mapping analysis. J Informetr (2017) 11(4):959–75. doi: 10.1016/j.joi.2017.08.007

[14] Anuwong A. Transoral endoscopic thyroidectomy vestibular approach: A series of the first 60 human cases. World J Surg (2016) 40(3):491–7. doi: 10.1007/s00268-015-3320-1 26546193

[15] Anuwong A Ketwong K Jitpratoom P Sasanakietkul T Duh QY. Safety and outcomes of the transoral endoscopic thyroidectomy vestibular approach. JAMA Surg (2018) 153(1):21–7. doi: 10.1001/jamasurg.2017.3366 PMC5833624 28877292

[16] Anuwong A Sasanakietkul T Jitpratoom P Ketwong K Kim HY Dionigi G. Transoral endoscopic thyroidectomy vestibular approach (TOETVA): indications, techniques and results. Surg Endosc (2018) 32(1):456–65. doi: 10.1007/s00464-017-5705-8 28717869

[17] Hüscher CS Chiodini S Napolitano C Recher A. Endoscopic right thyroid lobectomy. Surg Endosc (1997) 11(8):877. doi: 10.1007/s004649900476 9266657

[18] Gagner M. Endoscopic subtotal parathyroidectomy in patients with primary hyperparathyroidism. Br J Surg (1996) 83(6):875. doi: 10.1002/bjs.1800830656 8696772

[19] Choe JH Kim SW Chung KW Park KS Han W Noh DY. Endoscopic thyroidectomy using a new bilateral axillo-breast approach. World J Surg (2007) 31(3):601–6. doi: 10.1007/s00268-006-0481-y 17308853

[20] Kang SW Jeong JJ Yun JS Sung TY Lee SC Lee YS. Robot-assisted endoscopic surgery for thyroid cancer: experience with the first 100 patients. Surg Endosc (2009) 23(11):2399–406. doi: 10.1007/s00464-009-0366-x 19263137

[21] von Bohlen Und Halbach O. How to judge a book by its cover? How useful are bibliometric indices for the evaluation of “scientific quality” or “scientific productivity”? Ann Anat (2011) 193(3):191–6. doi: 10.1016/j.aanat.2011.03.011 21507617

[22] Aria M Alterisio A Scandurra A Pinelli C D’Aniello B. The scholar’s best friend: research trends in dog cognitive and behavioral studies. Anim Cognit (2021) 24(3):541–53. doi: 10.1007/s10071-020-01448-2 PMC8128826 33219880

[23] Nakajo A Arima H Hirata M Mizoguchi T Kijima Y Mori S. Trans-Oral Video-Assisted Neck Surgery (TOVANS). A new transoral technique of endoscopic thyroidectomy with gasless premandible approach. Surg Endosc (2013) 27(4):1105–10. doi: 10.1007/s00464-012-2588-6 PMC3599170 23179070

[24] Wang C Zhai H Liu W Li J Yang J Hu Y. Thyroidectomy: a novel endoscopic oral vestibular approach. Surgery (2014) 155(1):33–8. doi: 10.1016/j.surg.2013.06.010 23890962

[25] Russell JO Clark J Noureldine SI Anuwong A Al Khadem MG Yub Kim H. Transoral thyroidectomy and parathyroidectomy - A North American series of robotic and endoscopic transoral approaches to the central neck. Oral Oncol (2017) 71:75–80. doi: 10.1016/j.oraloncology.2017.06.001 28688695

[26] Kim HY Chai YJ Dionigi G Anuwong A Richmon JD. Transoral robotic thyroidectomy: lessons learned from an initial consecutive series of 24 patients. Surg Endosc (2018) 32(2):688–94. doi: 10.1007/s00464-017-5724-5 28726141

[27] Jackson NR Yao L Tufano RP Kandil EH. Safety of robotic thyroidectomy approaches: meta-analysis and systematic review. Head Neck (2014) 36(1):137–43. doi: 10.1002/hed.23223 23471784

[28] Ikeda Y Takami H Niimi M Kan S Sasaki Y Takayama J. Endoscopic thyroidectomy by the axillary approach. Surg Endosc (2001) 15(11):1362–4. doi: 10.1007/s004640080139 11727158

[29] Tae K Ji YB Song CM Ryu J. Robotic and endoscopic thyroid surgery: Evolution and advances. Clin Exp Otorhinolaryngol (2019) 12(1):1–11. doi: 10.21053/ceo.2018.00766 30196688 PMC6315214

[30] Singer MC Terris DJ. Robotic facelift thyroidectomy. Otolaryngol Clin North Am (2014) 47(3):425–31. doi: 10.1016/j.otc.2014.02.001 24882800

[31] Byeon HK Kim DH Chang JW Ban MJ Park JH Kim WS. Comprehensive application of robotic retroauricular thyroidectomy: The evolution of robotic thyroidectomy. Laryngoscope (2016) 126(8):1952–7. doi: 10.1002/lary.25763 26525822

[32] Chung EJ Park MW Cho JG Baek SK Kwon SY Woo JS. A prospective 1-year comparative study of endoscopic thyroidectomy via a retroauricular approach versus conventional open thyroidectomy at a single institution. Ann Surg Oncol (2015) 22(9):3014–21. doi: 10.1245/s10434-014-4361-7 25605517

[33] Sung ES Ji YB Song CM Yun BR Chung WS Tae K. Robotic thyroidectomy: Comparison of a postauricular facelift approach with a gasless unilateral axillary approach. Otolaryngol Head Neck Surg (2016) 154(6):997–1004. doi: 10.1177/0194599816636366 26980907

[34] Song CM Ji YB Kim KR Tae K. Robot-assisted excision of branchial cleft cysts using a postauricular facelift approach. Auris Nasus Larynx (2015) 42(5):424–7. doi: 10.1016/j.anl.2015.03.009 25863642

[35] Terris DJ Singer MC. Qualitative and quantitative differences between 2 robotic thyroidectomy techniques. Otolaryngol Head Neck Surg (2012) 147(1):20–5. doi: 10.1177/0194599812439283 22371342

[36] Karakas E Steinfeldt T Gockel A Westermann R Kiefer A Bartsch DK. Transoral thyroid and parathyroid surgery. Surg Endosc (2010) 24(6):1261–7. doi: 10.1007/s00464-009-0757-z 20033730

[37] Witzel K von Rahden BHA Kaminski C Stein HJ. Transoral access for endoscopic thyroid resection. Surg Endosc (2008) 22(8):1871–5. doi: 10.1007/s00464-007-9734-6 18163167

[38] Richmon JD Pattani KM Benhidjeb T Tufano RP. Transoral robotic-assisted thyroidectomy: a preclinical feasibility study in 2 cadavers. Head Neck (2011) 33(3):330–3. doi: 10.1002/hed.21454 20629089

[39] Miccoli P Materazzi G Berti P. Natural orifice surgery on the thyroid gland using totally transoral video-assisted thyroidectomy: report of the first experimental results for a new surgical method: are we going in the right direction? Surg Endosc (2010) 24(4):957–8. doi: 10.1007/s00464-009-0677-y 19707820

[40] AlRyalat SAS Malkawi LW Momani SM. Comparing bibliometric analysis using pubMed, scopus, and web of science databases. J Vis Exp (2019) 152:e58494. doi: 10.3791/58494 31710021

