# Supplementary material for: Global trends in research of endoscopic thyroidectomy from 2013 to 2022: a scientometric and visualization analysis
Source: Front Endocrinol (Lausanne). 2023 Aug 10;14:1199563. doi: 10.3389/fendo.2023.1199563 (PMC10449642; doi:10.3389/fendo.2023.1199563)
Supplement: Supplementary file 1 [file DataSheet_1.docx]

Supplementary Material

Supplementary Table S1 The top 10-cited documents in the research scope of endoscopic thyroidectomy with corresponding authors, sources, publication year, total citations, and total citations per year.

| **Title** | **Author*** | **Source** | **Year** | **Total Citations** | **Total Citations per Year** |
| --- | --- | --- | --- | --- | --- |
| Transoral Endoscopic Thyroidectomy Vestibular Approach: A Series of the First 60 Human Cases | Anuwong A | World Journal of Surgery 40 (3), pp.491-497 | 2016 | 272 | 34.00 |
| Safety and Outcomes of the Transoral Endoscopic Thyroidectomy Vestibular Approach | Anuwong A; Ketwong K; Jitpratoom P; Duh QY | Jama Surgery 153 (1), pp.21-27 | 2018 | 214 | 35.67 |
| Transoral endoscopic thyroidectomy vestibular approach (TOETVA): indications, techniques and results | Anuwong A; Sasanakietkul T; Jitpratoom P; Richmon JD | Surgical Endoscopy and Other Interventional Techniques 32 (1), pp.456-465 | 2018 | 134 | 22.33 |
| Impact of Postthyroidectomy Scar on the Quality of Life of Thyroid Cancer Patients | Choi Y; Lee JH; Kim YH; Roh MR | Annals of Dermatology 26 (6), pp.693-699 | 2014 | 123 | 12.30 |
| American Thyroid Association Statement on Remote-Access Thyroid Surgery | Berber E; Bernet V; Fahey TJ; Terris DJ | Thyroid 26 (3), pp.331-337 | 2016 | 117 | 14.63 |
| Trans-Oral Video-Assisted Neck Surgery (TOVANS). A new transoral technique of endoscopic thyroidectomy with gasless premandible approach | Nakajo A; Arima H; Hirata M; Natsugoe S | Surgical Endoscopy and Other Interventional Techniques 27 (4), pp.1105-1110 | 2013 | 117 | 10.64 |
| Thyroidectomy: A novel endoscopic oral vestibular approach | Wang CC; Zhai HN; Fahey TJ; Ding H | Surgery 155 (1), pp.33-38 | 2014 | 98 | 9.80 |
| Safety of robotic thyroidectomy approaches: Meta-analysis and systematic review | Jackson NR; Yao L; Fahey TJ; Kandil EH | Head and Neck-Journal for the Sciences and Specialties of the Head and Neck 36 (1), pp.137-143 | 2014 | 95 | 9.50 |
| Transoral thyroidectomy and parathyroidectomy - A North American series of robotic and endoscopic transoral approaches to the central neck | Russell JO; Clark J; Fahey TJ; Richmon JD | Oral Oncology 71, pp.75-80 | 2017 | 91 | 13.00 |
| Transoral robotic thyroidectomy: lessons learned from an initial consecutive series of 24 patients | Kim HY; Chai YJ; Fahey TJ; Richmon JD | Surgical Endoscopy and Other Interventional Techniques 32 (2), pp.688-694 | 2018 | 86 | 14.33 |

* First, second, third, and last authors

**Supplementary Table S2** Top 10 popular journals and cited journals. **A**) Top 10 popular journals regarding the number of related articles. **B**) Top 10 popular journals regarding the number of citations.

**A)** Top 10 popular journals.

| **Sources** | **Articles** | **2022 impact factor** | **2022 JCR partition** |
| --- | --- | --- | --- |
| Surgical Endoscopy and Other Interventional Techniques | 87 | 3.45 | Q1 |
| Surgical Laparoscopy Endoscopy & Percutaneous Techniques | 49 | 1.46 | Q2 |
| Gland Surgery | 44 | 2.16 | Q2 |
| Journal of Laparoendoscopic & Advanced Surgical Techniques | 42 | 1.77 | Q2 |
| Head and Neck-Journal for the Sciences and Specialties of the Head and Neck | 39 | 3.82 | Q1 |
| World Journal of Surgery | 26 | 3.28 | Q1 |
| Journal of Minimal Access Surgery | 15 | 1.02 | Q3 |
| Laryngoscope | 13 | 2.97 | Q1 |
| European Archives of Oto-Rhino-Laryngology | 12 | 3.23 | Q1 |
| Thyroid | 12 | 6.51 | Q1 |

**B)** Top 10 cited journals.

| **Sources** | **Articles** | **2022 impact factor** | **2022 JCR partition** |
| --- | --- | --- | --- |
| Surgical Endoscopy and Other Interventional Techniques | 2,384 | 3.45 | Q1 |
| World Journal of Surgery | 1,488 | 3.28 | Q1 |
| Surgery | 995 | 4.35 | Q1 |
| Surgical Laparoscopy Endoscopy & Percutaneous Techniques | 803 | 1.46 | Q2 |
| Head and Neck-Journal for the Sciences and Specialties of the Head aAnd Neck | 773 | 3.82 | Q1 |
| Laryngoscope | 743 | 2.97 | Q1 |
| Thyroid | 677 | 6.51 | Q1 |
| Journal of Laparoendoscopic & Advanced Surgical Techniques | 506 | 1.77 | Q2 |
| Annals of Surgical Oncology | 484 | 4.34 | Q2 |
| Gland Surgery | 431 | 2.16 | Q2 |

**Supplementary Table S3** The top 5 authors with the most times cited of their publications in the research scope of endoscopic thyroidectomy.

| **Author** | **Documents** | **Citations** | **Total link strength** |
| --- | --- | --- | --- |
| Anuwong A | 30 | 1,425 | 2738 |
| Kim HY | 43 | 1,149 | 2754 |
| Tufano RP | 45 | 1,041 | 2870 |
| Dionigi G | 48 | 1,036 | 2684 |
| Richmon JD | 12 | 566 | 1208 |


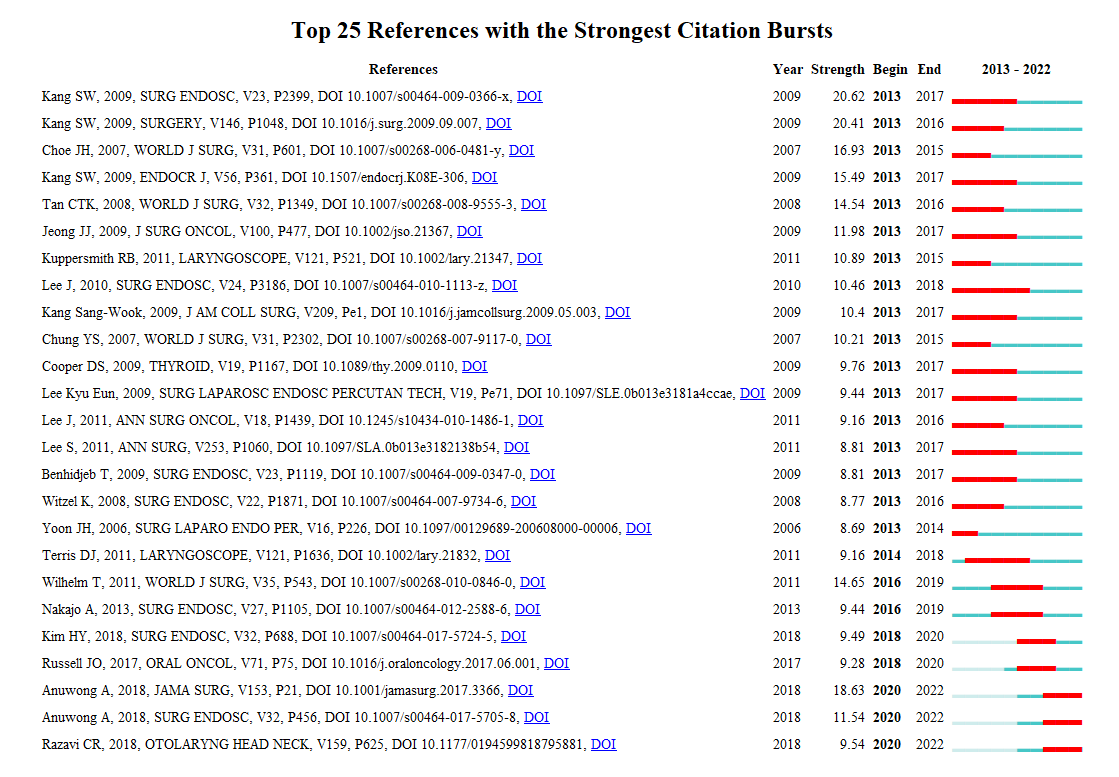


**Supplementary Figure S1** Top 25 references with the strongest citations burst.


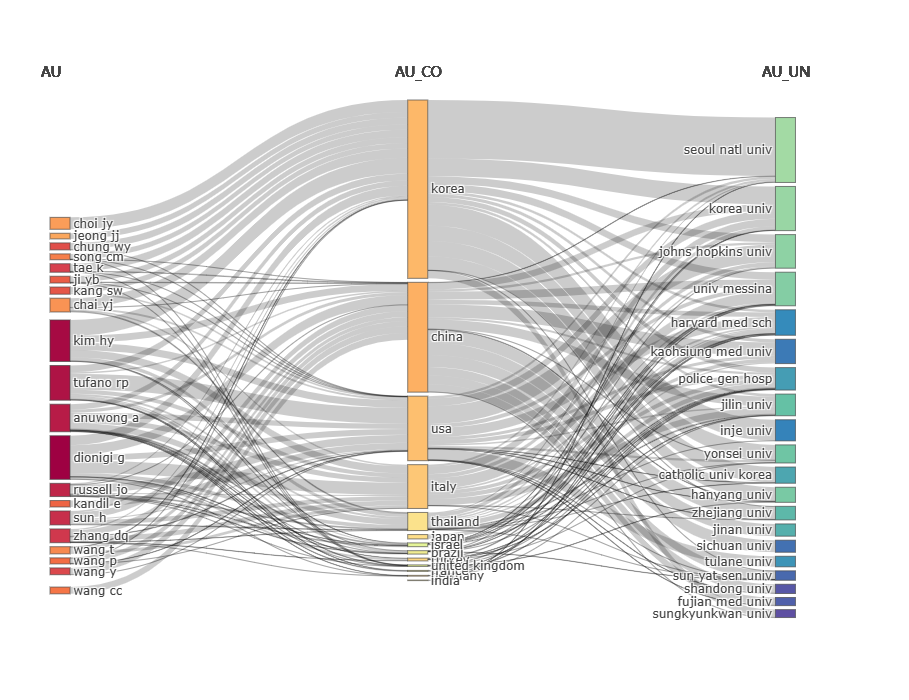


**Supplementary Figure S2** The three-field plot showing the top institutions (right), the most productive countries (middle), and the influential authors (left).


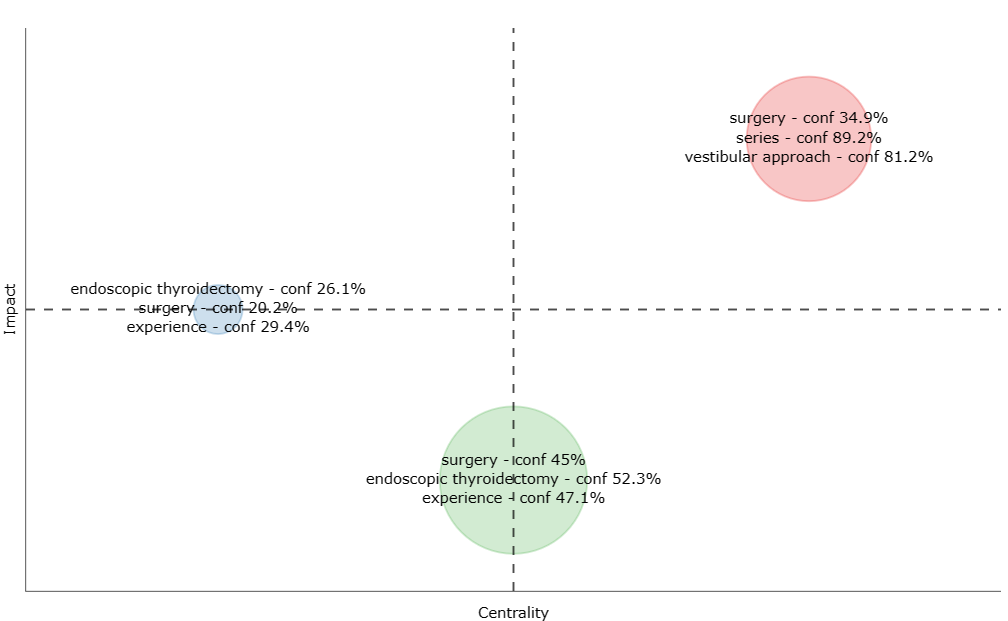


**Supplementary Figure S3** Keywords plus of four quadrants. (a) motor themes (first quadrant); (b) highly developed and isolated themes (second quadrant); (c) emerging or declining themes (third quadrant); (d) basic themes (fourth quadrant).


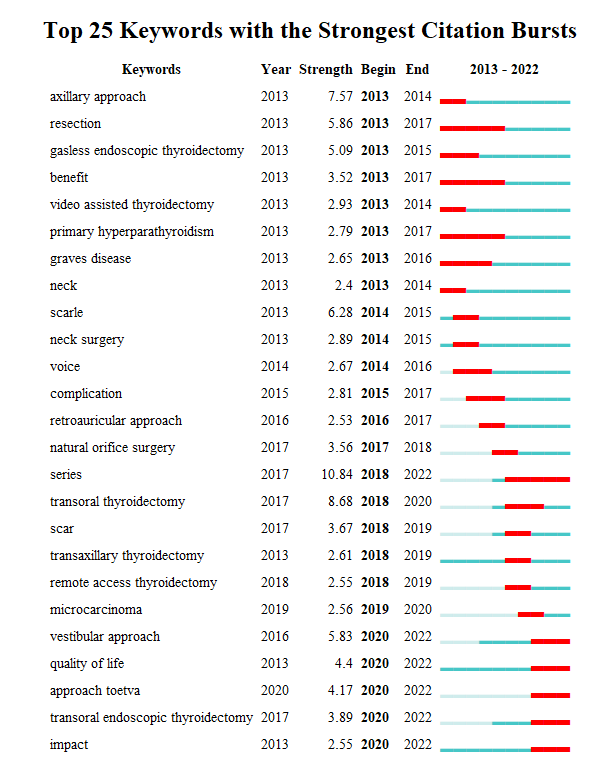


**Supplementary Figure S4** Top 25 keywords with the strongest citations bursts of this field.
